# Continental flood basalts do not drive later Phanerozoic extinctions

**DOI:** 10.1073/pnas.2303700120

**Published:** 2023-05-15

**Authors:** Michael J. Henehan, James D. Witts

**Affiliations:** ^a^Bristol Palaeobiology Research Group, School of Earth Sciences, University of Bristol, Bristol BS8 1RJ, United Kingdom

Green et al. (1) apply new statistical methods to a long-noted temporal correlation between mass extinctions and continental large igneous province (LIP) eruptions. Having concluded that the probability of said temporal coincidence by chance alone is extremely low, like others before them, Green et al. cite this correlation as evidence for causation. They then suggest that the temporal correlation of early Phanerozoic LIPs with mass extinctions implicates a similar volcanic driver for the later Cretaceous–Paleogene (K-Pg) extinction. While we do not dispute the link between early Phanerozoic mass extinctions and LIP volcanism, we suggest that it is counterproductive to draw equivalence between all episodes of LIP volcanism in this way, when doing so ignores clear differences in the circumstances and observed effects of individual LIPs.

Earth system scientists have long recognized that the rise of pelagic marine calcifiers fundamentally changed the way Earth responds to CO_2_ injection (e.g. ref. 2). The mid-Mesozoic emergence of a readily dissolvable deep ocean CaCO_3_ reservoir meant for perturbations on timescales of LIP volcanism surface ocean carbonate saturation (Ω) could henceforth be maintained by carbonate compensation, reducing the threat to marine ecosystems from fluctuating Ω. Such later Phanerozoic resilience meant when LIP volcanism injected ∼10,000 Pg C at the Paleocene-Eocene Thermal Maximum (3) carbonate compensation buffered surface Ω and—excepting among deep-sea benthic foraminifera—large-scale marine extinctions were avoided. Similarly, CO_2_ release at the onset of Deccan emplacement drove the Late Maastrichtian Warming Event, but carbonate dissolution buffered surface ocean Ω (4) and marine communities show no elevated extinction rates at this time (e.g., refs. 5 and 6).

**Fig. 1.**
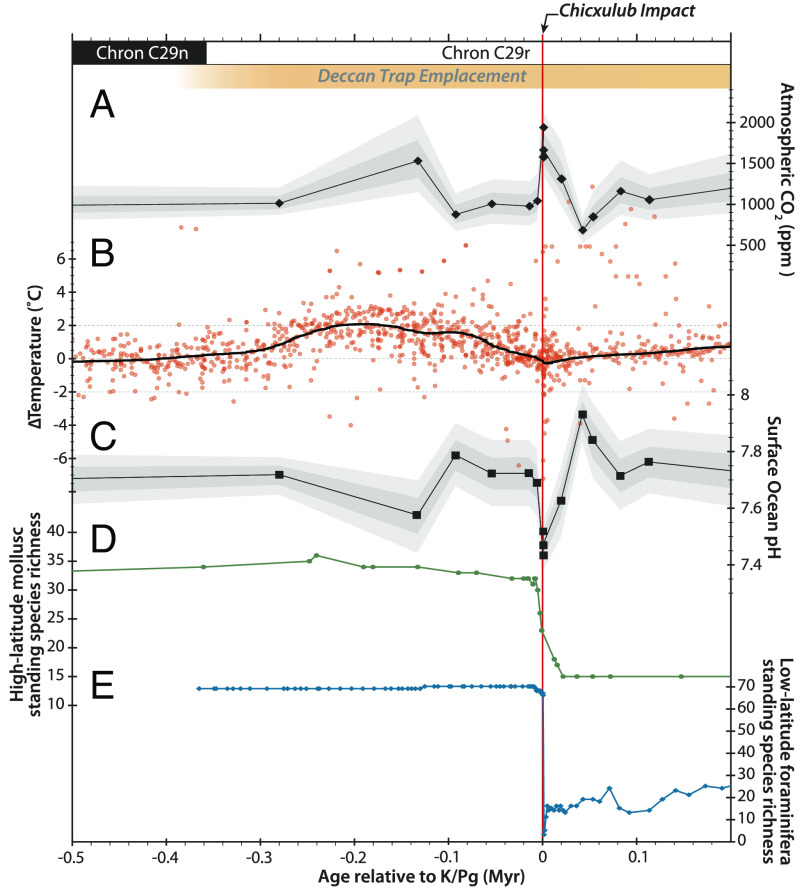
With only a transient excursion in atmospheric CO_2_ (panel *A*, from ref. 7), global temperature (panel *B*, from ref. 8), and surface ocean pH (panel *C*, from ref. 7) during the onset of Deccan volcanism, and conditions returning to preevent values by the time of the Chicxulub impact, there is little evidence for Deccan outgassing contributing to the Cretaceous–Paleogene mass extinction. Even the high-latitude fossil record cited by ref. 1 as showing gradual extinction has been shown by higher resolution sampling panel *D*, from ref. 6 to be a product of the Signor–Lipps effect, and in fact, this high-latitude location shows no more evidence for gradual extinction than low latitude diversity data panel *E*, from refs. 5 and 9. Note, absolute pH and pCO_2_ values plotted here differ from ref. 7, as calculations here use updated seawater equilibrium constants.

Having overlooked the fundamentally different biogeochemical boundary conditions upon which Deccan CO_2_ release was superimposed relative to earlier Phanerozoic LIPs, Green et al. (1) cite putative gradual extinction at high latitudes as support for Deccan-induced “stress” in the run-up to the K-Pg. This purported high-latitude gradual extinction has however been shown to be an artifact of poor sampling (6). Furthermore, increasing stress is belied by atmospheric CO_2_ (7) and global temperature (8) returning to pre-Deccan levels prior to the Chicxulub impact (Fig. 1) and is inconsistent with sedimentological (4) and paleontological (e.g. refs. 5 and 6) evidence. Indeed, the idea that ecosystems sustained by organisms with lifespans of days to months can be “stressed” for hundreds of thousands of years without evolving resilience seems inconsistent with evolutionary theory.

In sum, we implore the community to move beyond implicating causality from temporal coincidence, and instead, to focus on how, mechanistically, individual LIPs interacted with their specific boundary conditions to drive specific environmental and macroevolutionary responses. While meaningful research questions about the rate and scale of perturbation required to prompt a mass extinction remain, ultimately, a causal link between any geological phenomenon and mass extinction cannot be made by comparing unconnected events hundreds of millions of years apart. Instead, feasible, model-supported mechanisms for how a given event could precipitate ecological collapse are required—mechanisms that for the Chicxulub impact exist, in the form of ocean acidification (7) and impact winter (10).
